# 270. RhD negative Blood Type is Associated with Higher Levels of *Babesia microti* Parasitemia and May Be a Useful Point-of-Care Biomarker in Human Babesiosis

**DOI:** 10.1093/ofid/ofad500.342

**Published:** 2023-11-27

**Authors:** Reshma George, Michael D Lum, Andreas Kalogeropoulos, Eric Spitzer, Luis A Marcos

**Affiliations:** Stony Brook University Hospital, Seaford, New York; Stony Brook University Hospital, Seaford, New York; Stony Brook University, Stony Brook, New York; Stony Brook Medicine, Stony Brook, New York; Stony Brook University Hospital, Seaford, New York

## Abstract

**Background:**

There is a growing need to identify novel point-of-care biomarkers that correlate with clinical presentation in the study of babesiosis. Recently, Jajosky et al (2023, PMID: 36696414) demonstrated that in patients with babesiosis, lack of expression of Rhesus (RhD) factor antigen was associated with elevated levels of parasitemia, a potential risk factor for severe disease. The goal of this study is to add to these findings by analyzing a babesiosis cohort with varying ABO and RhD factor antigens and assessing for any correlation with increased disease severity.

**Methods:**

A total of 52 cases of confirmed babesiosis at Stony Brook University Hospital from 2008 to 2014 were reviewed. Maximum parasitemia (Mp), hospital length of stay (LoS), Intensive Care Unit (ICU) admission, and need for blood transfusion or exchange transfusion (XT) were compared amongst patients with varying ABO and RhD antigen status. Mp and LoS were compared among ABO and RhD groups with Analysis of Variance (ANOVA) after log transformation. Logistic regression was used to compare rates of ICU admission and XT among groups. Fisher’s exact test was used to assess requirement for RBC transfusion between RhD groups.

**Results:**

Patient characteristics and clinical information were recorded (Table 1). There was no significant difference in LoS, ICU admission, or XT among ABO groups (P = 0.43, P = 0.76, P = 0.10, Table 2) and among RhD groups (P = 0.094, P =0.28, P = 0.72, Table 2). While no difference was seen in Mp among ABO groups (P = 0.58, Fig1A), RhD- patients were more likely to have a higher Mp when compared to RhD+ patients (median of 6.7% vs 1.6%; P = 0.036, Fig 1B, Table 2). RhD- patients had greater requirement for blood transfusions compared to RhD+ patients (P = 0.03, Table 2).

Table 1

**Figure:**
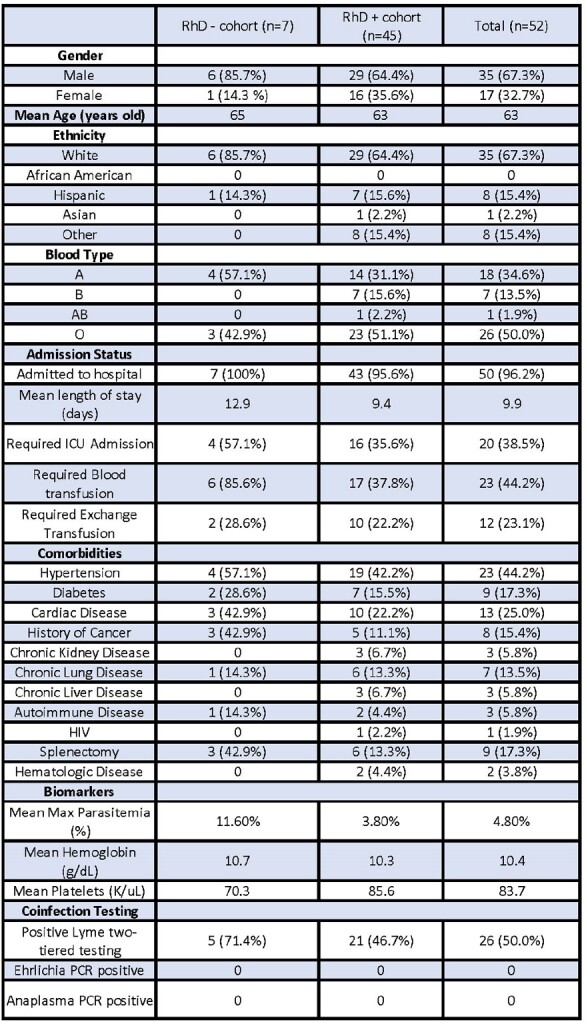

Summary of patient demographics and clinical data among RhD groups versus total sample population.

Table 2

**Figure:**
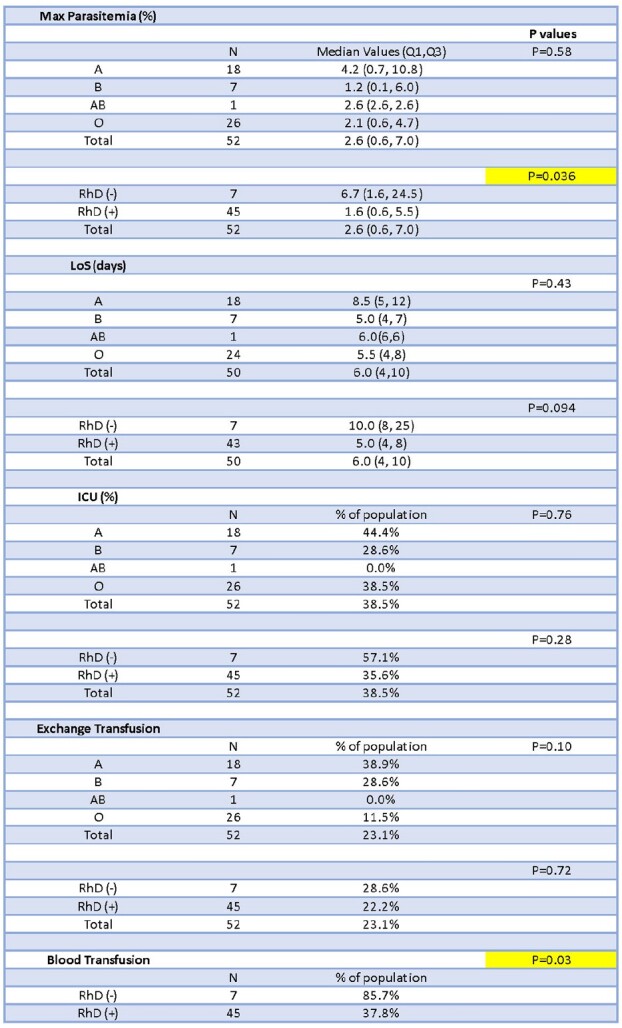

Analysis of Maximum parasitemia, hospital length of stay (LoS), Intensive Care Unit (ICU) admission, and need for blood transfusion or exchange transfusion among ABO/RhD groups.

Figure 1

**Figure:**
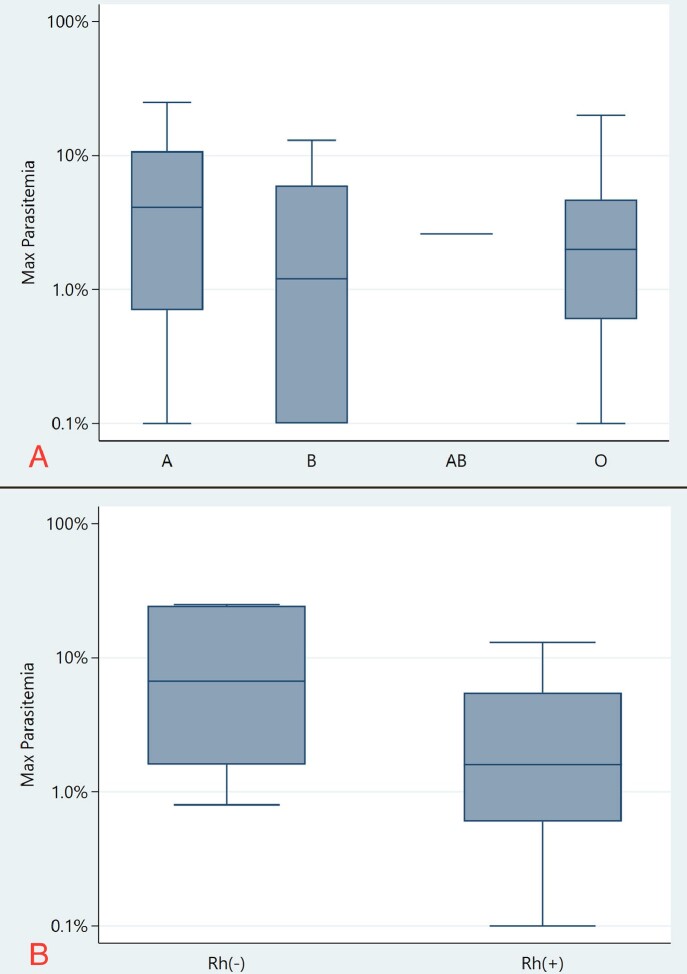

(A)Box plot analysis of median maximum parasitemia (Mp) among ABO groups. Blood type A had the highest Mp at 4.2% with an interquartile range of 10.1%. Blood type B had the lowest Mp at 1.2% with an interquartile range of 5.9%. Only one patient had blood type AB with a median Mp of 2.6%. Blood type O had a median Mp of 2.1% with an interquartile range of 4.1%. There was no statistically significant difference in Mp among ABO groups (P = 0.58, table 2). (B) Box plot analysis of median Mp among RhD groups. RhD- had the highest median Mp at 6.7% with an interquartile range of 22.9%. RhD+ had the lowest median Mp at 1.6% with an interquartile range of 4.9%. There was a statistically significant difference in Mp among RhD groups (P = 0.036, table 2).

**Conclusion:**

Our study suggests that RhD- patients may be at increased risk of severe babesiosis due to the greater parasite burden. Although there were no differences in LoS or ICU admission, the RhD- patients received more blood transfusions. Our findings support those of Jajosky et al and suggest that further studies should evaluate the potential of RBC antigens to impact the clinical course of babesiosis.

**Disclosures:**

**All Authors**: No reported disclosures

